# Pathological lung tissue changes in common infectious diseases

**DOI:** 10.6026/973206300220529

**Published:** 2026-01-31

**Authors:** Amit Nampalliwar, Sachin Pundlikrao Ambirwar, Swati Narayan Khandale, Prashant Uttam Sasane, Sheetal Suryakant Chavan, Chandreshwar Prasad Sinha

**Affiliations:** 1 Department of Pathology (Roga Nidan & Vikriti Vigyana), Government Ayurved College & Hospital, Bilaspur (C.G.), Chhattisgarh, India; 2 Department of Pharmacology, Lokmanya Tilak Municipal Medical College and General Hospital, Mumbai, India; 3 Department of Physiology (Kriya Sharira), Institute of Teaching and Research in Ayurveda, Jamnagar, Gujarat, India; 4 Department of Internal Medicine(Kayachikitsa), All India Institute of Ayurveda, Goa, India; 5 Department of Pathology(Roga Nidan & Vikriti Vigyana), Ayurved Seva Sangh's, Ayurved Mahavidyalaya, Ganeshwadi, Panchavati, Nashik, Maharashtra, India; 6 Department of Internal Medicine(Kayachikitsa), Sri. N.P.A. Government Ayurved College, Raipur (C.G.), Chhattisgarh, India

**Keywords:** Infectious lung diseases, histopathology, pneumonia, tuberculosis, fungal infections, viral pneumonia

## Abstract

Infectious diseases of the lung remain a major cause of morbidity and mortality worldwide and their diagnosis often relies on characteristic histopathological patterns. Hence, this retrospective observational study analysed 90 archived lung biopsy, resection and autopsy specimens to compare pathological changes associated with bacterial, viral, fungal and tuberculous infections. Bacterial infections were the most common. They showed predominant acute inflammation and consolidation. Tuberculosis and fungal infections were characterized by granulomatous inflammation and caseous necrosis. Viral infections demonstrated alveolar damage. Distinct histopathological profiles correlated with specific infectious etiologies and clinical outcomes, underscoring the diagnostic and prognostic value of microscopic examination in infectious lung diseases.

## Background:

Pneumonia and other infectious lung diseases are commonly known to be a significant source of morbidity and mortality across the globe, especially in the low- and middle-income nations. The histopathological examination is very important in diagnosis when there is a limitation or inconclusive microbiological confirmation. Cytopathology and histopathology are important problem-solving techniques in lower respiratory tract infections [1]. Pulmonary infections exhibit broad range of morphological patterns which are based on host anatomy, immunological reaction and environmental variables [2]. The typical symptoms of bacterial pneumonias are inflammation of acute nature, neutrophilic inflammation and consolidation of the alveols, which are repeatedly observed in literature of classical pathology and in recent researches [3]. In developing areas, tuberculosis is still among the major diseases causing chronic pulmonary infection. It has typical histological features, which consist of granulomatous inflammation, Langhans giant cells and caseous necrosis. The following features are the focus of pathological diagnosis [4]. Viral pulmonary infections have been the focus of new interest following the outbreak of SARS-CoV-2. Typical histopathological changes that were typical including diffuse damage in the alveolas, formation of hyaline membrane as well as microvascular injury were regularly documented [5]. The diagnostic difficulty to detect viral related acute respiratory diseases syndrome has been noted in postmortem studies where it is hard to detect certain cases especially when using autopsy samples [6]. These histological characteristics are still critical in the differentiation of viral pneumonias and bacterial and mycobacterial pneumonias [5].

The experimental models also show that Mycobacterium tuberculosis causes tightly regulated immunopathological events resulting in the formation of granuloma and disease progression [7]. Moreover, histological examination is also an essential step in interpreting the multiplex molecular test outcomes, especially in postmortem and minimally invasive tissue testing [8]. According to recent studies based on autopsy, it is possible that pulmonary patterns of infection are changing in the post-COVID-19 period. It has been observed that there are significant changes in the lung lesions during and after the pandemic [9]. The necessity of renewed clinicopathological correlation in modern practice is also supported by such epidemiological observations as the late re-emergence of Mycoplasma pneumoniae after the end of pandemic restrictions [10]. In the diagnostic methods, the use of combined metagenomic next- generation sequencing and histopathological analysis is shown to be more effective in identifying pathogens involved in pulmonary infections [11]. Future uses of automated histological interpretation may involve the emergent computational pathology methods, such as machine-learning-based detection of pneumonia subphenotypes [12]. In spite of these changes, the available literature is rather individual-pathogen oriented and there is little information on examining and comparing histopathological patterns of bacteria, viruses, fungi and mycobacteria in the same group of participants [13]. Moreover, clinicopathological correlation has been useful in other pulmonary diseases justifying its applicability in infectious lung pathology studies [14]. Thus, the analysis of histopathological alterations in various infectious etiologies and correlation with clinical outcomes is incomplete, which can be improved to increase the accuracy of diagnosis and prognosis. This work contributes greatly towards the understanding of histopathological patterns in the common infectious lung diseases. Therefore, it is of interest to evaluate histopathological changes on lung tissue compared to other prevalent incorporative etiologies.

## Methodology:

## Study design and setting:

This was tertiary care hospital/medicine college research that was a retrospective observational study. It entailed the review of archived lung biopsy, resection and autopsy specimens and their respective histopathology reports which were all previously diagnosed with infectious pathology. Histopathological patterns which are the outcomes of the most common infectious diseases of the lungs were recorded and compared as the major aim.

## Sample size and selection criteria:

The investigation targeting 90 cases was carried out. Lung tissue specimens were selected upon the criteria of appropriately preserved slides and complete clinical and pathological record. Only less than two-year-old slides were used so that the stability of staining and integrity of tissues could be the most optimal ones. These are the slides that are known to maintain their morphological and staining characteristics quite well. The inclusion criteria were first and foremost cases with histologically confirmed infectious lesions in the lungs and also accompanied with full clinical and pathology reports. Poorly preserved, faded, autolysed or inadequately prepared tissue sections were not used. Areas with completely non-infectious pathology, e.g. neoplastic lesions without an infection, were not included either. Also, those cases, whose clinical or pathological data were not complete, were excluded.

## Data collection procedures:

The histopathological examination involved the retrieval of archived hematoxylin and eosin (H & E) stained slides on all the 90 cases and re-examination of the slides using light microscopy. Initial diagnoses were revisited by corresponding histopathology reports and microscopic results were augmented. In the process of slide evaluation, some parameters were recorded. These were the nature of inflammation (acute or chronic and granulomatous). It was also observed to have necrosis, oedema, haemorrhage and fibrosis. The recorded predominant cellular infiltrates were also the neutrophils, lymphocytes, plasma cells, macrophages and multinucleated giant cells. Other morphological characteristics such as abscess formation, cavitation, bronchial involvement, consolidation, alveolar damage and granuloma formation were also noted. In case of availability, special stains described in the initial reports were taken into account. These were stains of Ziehl-Neelsen Mycobacterium tuberculosis and PAS or GMS fungus. Medical records provided the clinical data of each case. This information involved the age, sex, infection type (bacterial, viral, fungal or tuberculosis), clinical diagnosis and radiological results. Clinical outcomes (treated, complicated, or mortal) and the details of the treatment were also documented. The history pathological features were correlated with these clinical parameters to identify the patterns and association among the different infectious categories.

## Data analysis:

Data gathered in all the sources were summarized in tables and are subsequently analyzed using descriptive statistical techniques. Demographic variables and the histopathological features were presented in frequencies and percentages. The chi-square test or a Fisher, exact test was done to test the difference in the categories of infectious diseases by depending on the case at hand and a p-value of less than 0.05 was considered to be the statistically significant one. Any statistical and data management was performed with the assistance of Microsoft Excel.

## Ethical considerations:

The study's ethical approval was obtained before the data evaluation. As the study was based only on archived specimens and clinical and pathology records, there was no need for direct patient contact. All identifying information was anonymised to maintain confidentiality and the data were solely for scholarly and research objectives.

## Results:

**Figure 1:**
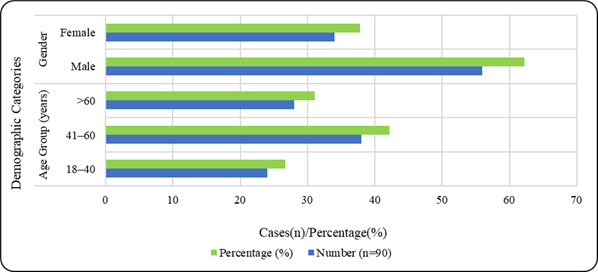
Demographic distribution of cases

**Figure 2:**
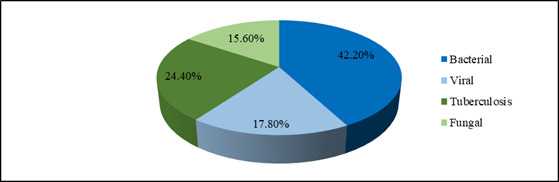
Distribution of Infectious Etiologies

Analysis was done on 90 cases of infectious lung pathology. The patients' ages varied from 18 to 82, with a mean age of 48.6 years. The age range of the majority of patients was 41-60 years old. There was a male predominance within the study population, with 56 patients (62.2%) being male and 34 (37.8%) being female. Table 1 shows the age groups and gender proportions of the research population's demographic distribution. Table 1 shows that the 41-60-year age group constituted the largest proportion of cases and males were more frequently affected than females. The graphical representation of the demographic characteristics is shown in Figure 1. Figure 1 clearly illustrates the the greater number of middle-aged people and more significant participation of males in the study group. Bacterial infections were the prevalent cases (38 cases) representing 42.2% of the total 90 cases. Tuberculosis accounted for 22 cases (24.4%), viral infections for 16 cases (17.8%) and fungal infections for 14 cases (15.6%). The proportional distribution of different infectious etiologies is shown in Figure 2. Bacterial infections formed the largest group, followed by tuberculosis. Fungal and viral infections were comparatively less frequent. Figure 2 demonstrates that bacterial infections formed the largest group, followed by tuberculosis, while fungal and viral infections were comparatively less frequent. Table 2 further summarises the frequency and percentage of each infectious etiology included in the study.

**Figure 3:**
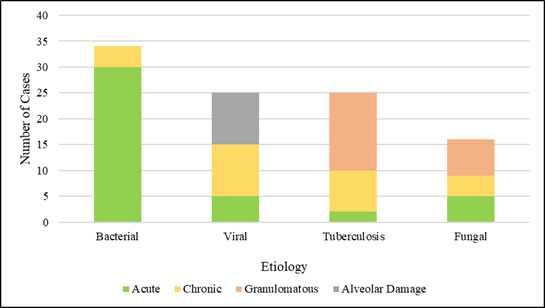
Key histopathological patterns across the study

As seen in bacterial infections constituted the highest proportion, whereas fungal infections were the least represented among the study cases. Acute inflammation dominated the findings and was observed in 42 cases (46.7%), primarily in bacterial infections. Chronic inflammation was present in 26 cases (28.9%), while granulomatous inflammation was noted in 22 cases (24.4%), all associated with tuberculosis and fungal infections. Necrosis was present in 30 cases (33.3%), including 20 cases (22.2%) showing caseous necrosis exclusively in tuberculosis. Oedema was observed in 48 cases (53.3%), haemorrhage in 32 cases (35.6%) and fibrosis in 18 cases (20%), particularly in chronic granulomatous lesions. Bacterial pneumonia had a high rate of neutrophil-rich infiltrates (44 cases (48.9%)). A preponderance of lymphocytes was observed in 28 cases (31.1%) usually in viral infections. In 22 cases (24.4%), macrophage infiltration was observed and 18 cases (20%), primarily in the case of tuberculosis and fungi, multinucleated giant cells. In 12 cases (13.3%), abscesses had been formed, mostly related to infections with Staphylococcus aureus. There was a case of 10 cavitations (11.1%), predominantly in tuberculosis. In 38 cases (42.21%) it was noted that consolidation was present, whereas in 10 cases of the virus (11.1%) there was evidence of alveolar damage with hyaline membranes. A full summary of all key histopathology features that were found in all the cases of the study has been summarized in Table 3. Table 3 shows that the acute inflammation, edema and consolidation were the most common histopathological changes and cavitation and alveolar damage were observed relatively rarely. Figure 3 illustrates the pattern of distribution of key histopathological patterns in the various infectious etiologies in a graphical manner. It is shown in Figure 3 that acute inflammation was the most common in infections by bacteria, granulomatous in infectious diseases caused by tuberculosis and fungi and damage to the alveoli in infections caused by viruses.

**Figure 4:**
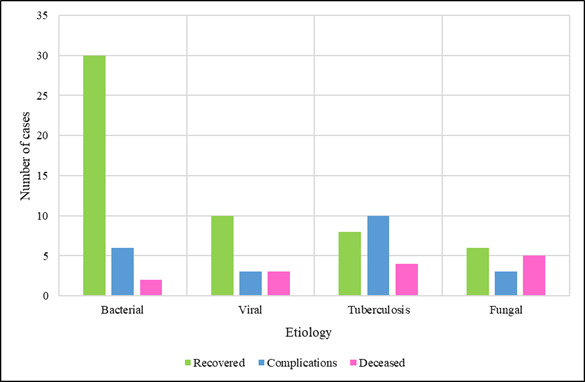
Clinical outcome comparison among infection types

Chi-square analysis was conducted on the important morphological variables in order to identify any association of specific infectious etiologies with different histopathological findings. Table 4 results indicated statistically significant pathogen-specific tissue response associations. Bacterial infections were also found to exhibit a high correlation with acute inflammation with 30 out of 38 cases (78.9) exhibiting acute inflammatory patterns (χ^2^ = 41.82, p < 0.001). Viral infections were already also considerably correlated with alveolar damage which was observed in 10/16 cases (62.5) (χ^2^ = 46.55, p < 0.001). Tuberculosis showed a highly significant association with granulomatous inflammation, present in 15 of 22 cases (68.2%) (χ^2^ = 84.77, p < 0.001) and with caseous necrosis, seen in 20 of 22 cases (90.9%) (χ^2^ = 102.14, p < 0.001). Fungal infections demonstrated significant correlations with both granulomatous inflammation (50%) and giant cell formation (χ^2^ = 27.13, p < 0.001). These findings confirm that each major infectious group displays a distinct histopathological profile with statistically significant etiological associations. Table 5 shows clear, statistically significant associations between each infectious etiology and its characteristic histopathological features, confirming distinct morphological patterns for bacterial, viral, tuberculous and fungal infections. Among the 90 cases, 54 patients (60%) recovered, 22 patients (24.4%) developed complications and 14 patients (15.6%) died during the course of illness. Poor outcomes were more frequently associated with tuberculosis and severe viral pneumonias. The distribution of clinical outcomes among the study population is summarised in Table 5. Table 4 indicates that the majority of patients recovered, although a significant proportion experienced complications or mortality, particularly in tuberculosis and viral infection groups. Figure 4 presents a comparative visualisation of recovery, complication and mortality rates across the major infection types. The Figure 4 shows that bacterial infections had the highest recovery rates, while tuberculosis and fungal infections demonstrated the greatest proportion of complications and fatalities.

## Discussion:

This study analyzed the histopathological patterns of the lung diseases associated with common infections using 90 retrospectively analyzed cases. The findings illustrate clear morphological variations of each of the bacterial, viral, fungal and tuberculous infections hence the significance of histopathology in differentiating and prognosticating. The demographic data showed that there were male middle-aged people that dominated and this is accurate to the previously reported patterns of respiratory infections [1]. The greatest proportion of the cases was attributed to the bacterial infections (42.2), then tuberculosis, viral infections and fungal diseases. The patterns of the diseases are similar to the entire world whereby bacterial pneumonia remains a major cause of infectious lung diseases [15]. The study has shown that tuberculosis has an overwhelming and persistent weight in developing regions with its prevalence of 24.4%. We found the same results as Djannah *et al.* [4], who also found high rates of granulomatuous and caseous lesions in people who reside in TB-infested regions. On a histological level, the most frequent patterns were acute inflammation, edema and consolidation, mainly in bacterial infections. They are features that fit very well with the traditional morphological features of acute bacterial pneumonia characterized by the presence of neutrophilic exudation and alveolar filling [3]. Viral infections revealed damage to the alveolar structures with the presence of hyaline membranes that are typical for diffuse alveolar injury as described in COVID-19 and other viral pneumonitides [6]. The study's result that alveolar damage was present in 11.1% of the cases is very close to that of the ARDS studies based on autopsy, where epithelial injury and edema was the major pathological changes. Granulomatous inflammation and caseous necrosis were the only features that appeared in tuberculosis and some fungal infections in our group. These findings are very similar to those of Kolloli *et al.* [7], who found a strong granulomatous response in experimental TB models. Similarly, Muthureddy *et al.* [9] pinpointed granulomas and giant cells as main features of chronic infectious lung lesions in their post-COVID autopsy series.

The occurrence of the cavitation in 11.1% of the cases is consistent with the established TB pathology thereby cementing the diagnostic position of the histomorphology in the differentiation of chronic infectious processes. One of the main results of the current study was the presence of pathological crossover in mixed infectious states. It was less common, yet there were some cases of intertwined characteristics of chronic inflammation over imposed on the acute patterns. The results are in line with the idea of complex pulmonary infection introduced by Ritter *et al.* [8]. This brings in the necessity of multimodal diagnostics. Moreover, Yang *et al.* [11] Support the use of histopathology with other procedures such as molecular tests, which is consistent with our methodological approach to the use of routine and specialized staining to ensure proper diagnosis. Clinical outcome analysis indicated that 60 percent of the patients healed but the primary cause that resulted into complications and death was tuberculosis and viral infections. These findings are in line with those of Waldeck *et al.* [16], who observed adverse outcomes in viral-bacterial coinfections and delays in the diagnosis of TB-related pneumonia. Moreover, linking severe histopathological features such as alveolar destruction and necrosis to death indicates the tissue-based assessment as a source of prognostic information [13]. Although the research is robust in many aspects, it is limited in several ways. Firstly, the retrospective nature of the study limits the researchers' ability to control clinical variables and standardization of diagnostics. Secondly, the study only included cases that had sufficient archival material; hence, there might be a selection bias. Thirdly, microbiological confirmation was not performed for all the cases, thus limiting the direct relationship between pathogen type and histopathology. Moreover, the slides that were taken into account were not older than two years; this was done to preserve staining quality, but these samples may not represent the full range of long-term archived samples. Finally, the study focused on one single center and the findings may not apply to a larger population with different pathogen distribution patterns. Future researchers must pay attention to the integration of histopathology with advanced molecular techniques such as next-generation sequence, which is highly precise in diagnosing pulmonary infections [17]. Prospective multicenter research that uses a significant sample size is required to verify the relationships in this case. The use of the machine-learning method to identify histopathological subphenotypes, as applied by Yu *et al.* [12], may take a step further to demystifying the diagnostic classification and the forecasting. In addition to it, research of the periodic variation of the infectious processes, especially during the post-COVID period, can assist in the detection of the emerging pulmonary pathology profiles [10]. In the current study, the use of histopathology is justified as a tool in the process of diagnosing infectious lung diseases. It also demonstrates the varying modifications in tissue structures associated with the various etiological groups. This piece of work can open new possibilities in the existing literature and can contribute to the preservation of histopathological assessment as an inseparable component of diagnostic teams used in different specialties.

## Conclusion:

This paper demonstrated typical pathological characteristics in infectious conditions affecting the lungs that possess clear histopathological characteristics which are closely linked to causes and prognosis. The inflammation was predominantly acute when there was bacterial infection, granulomatous when there was tuberculosis and other fungal infections as well as diffuse alveolar when there were viral pneumonias. The diagnosis and prognosis of the disease were high because the pathological changes were severe and finally resulted in higher morbidity and mortality rates. The microscopic diagnosis and management of infectious lung diseases continue to need the incorporation of both microscopic findings and clinical data to provide the needed diagnosis and treatment.

## Figures and Tables

**Table 1:** Demographic profile of the study population

**Variable**	**Category**	**Number (n=90)**	**Percentage (%)**
Age Group (years)	18-40	24	26.7
	41-60	38	42.2
	>60	28	31.1
Gender	Male	56	62.2
	Female	34	37.8

**Table 2:** Frequency of different infectious etiologies

**Etiology**	**Number of Cases (n=90)**	**Percentage (%)**
Bacterial	38	42.2
Viral	16	17.8
Tuberculosis	22	24.4
Fungal	14	15.6

**Table 3:** Summary of histopathological findings

**Feature**	**Number (n=90)**	**Percentage (%)**
Acute inflammation	42	46.7
Chronic inflammation	26	28.9
Granulomatous inflammation	22	24.4
Necrosis	30	33.3
Caseous necrosis	20	22.2
Hemorrhage	32	35.6
Edema	48	53.3
Fibrosis	18	20
Neutrophil-rich infiltrates	44	48.9
Lymphocyte-rich infiltrates	28	31.1
Giant cells	18	20
Cavitation	10	11.1
Abscess formation	12	13.3
Consolidation	38	42.2
Alveolar damage	10	11.1

**Table 4:** Chi-square analysis of associations between infectious etiology and key histopathological features

**Histopathological Feature**	**χ^2^ Value**	**df**	**p-value**	**Significant Association**
Acute inflammation	41.82	3	<0.001	Bacterial infections
Chronic inflammation	18.44	3	<0.001	Viral & fungal groups
Granulomatous inflammation	84.77	3	<0.001	TB & fungal infections
Caseous necrosis	102.14	3	<0.001	Tuberculosis only
Alveolar damage	46.55	3	<0.001	Viral infections
Giant cells	27.13	3	<0.001	TB & fungal infections

**Table 5:** Clinical outcome distribution

**Outcome**	**Number (n=90)**	**Percentage (%)**
Recovered	54	60
Complications	22	24.4
Deceased	14	15.6

## References

[1] File TM-Jr, Ramirez JA (2023). N Engl J Med..

[2] Febbo J (2022). Radiol Clin North Am..

[3] Gagiannis D (2022). Am J Clin Pathol..

[4] Djannah F (2022). Ann Med Surg (Lond)..

[5] Deshmukh V (2021). J Clin Pathol..

[6] de Araújo LJT (2023). Appl Immunohistochem Mol Morphol..

[7] Kolloli PM (2024). Int J Mol Sci..

[8] Ritter JM (2021). Clin Infect Dis..

[9] Muthureddy Y (2023). J Clin Diagn Res..

[10] Sauteur P (2024). Lancet Microbe..

[11] Yang L (2024). Microbiol Spectr..

[12] Yu KH (2020). J Am Med Inform Assoc..

[13] Eshaghi S (2025). Open Forum Infect Dis..

[14] Ramniwas (2022). Indian J Chest Dis Allied Sci..

[15] Ma Y (2025). Infection..

[16] Waldeck F (2025). Int J Infect Dis..

[17] Sarfo JO (2023). BMC Pediatr..

